# How Far Has Radiofrequency Thermocoagulation Come Along as a Treatment Procedure in Treating Trigeminal Neuralgia Patients?

**DOI:** 10.7759/cureus.40311

**Published:** 2023-06-12

**Authors:** Stephen D Howard, Varun Soti

**Affiliations:** 1 Physical Medicine and Rehabilitation, Lake Erie College of Osteopathic Medicine, Elmira, USA; 2 Pharmacology and Therapeutics, Lake Erie College of Osteopathic Medicine, Elmira, USA

**Keywords:** postoperative pain relief, procedural advancement, effectiveness and safety, radiofrequency thermocoagulation, trigeminal neuralgia

## Abstract

Trigeminal neuralgia (TN) refers to sudden shooting pain in areas innervated by trigeminal nerves originating from the Gasserian ganglion. Physicians initially manage it by prescribing drugs, such as carbamazepine. Surgical intervention is the next best option if patients do not respond to drug treatments. These procedures include microvascular decompression, rhizotomy, balloon compression, and gamma knife surgery. However, less optimal patient outcomes, recurrences, adverse effects, and high costs have necessitated alternative surgical interventions to treat such patients. Radiofrequency thermocoagulation (RFT) has emerged as a minimally invasive, safer, and effective surgical option in treating TN patients. Despite research showing RFT’s safety and effectiveness, neurosurgical healthcare providers do not frequently use it to treat TN patients. Lack of universal standardized protocol, and minimal awareness of its efficacy in specific cohorts, such as geriatric patients, may lead to RFT underutilization. Hence, this review highlights RFT's advancement as a robust alternative to traditional surgical approaches in treating TN patients. In addition, it identifies RFT’s areas of improvement and its safety and effectiveness in treating elderly TN patients. We followed the Systematic Reviews and Meta-Analyses guidelines for systematic reviews and conducted a literature search between July 2022 and March 2023. Our findings indicate that RFT has evolved significantly over the last decade and a half as a minimally invasive and effective treatment procedure for TN patients. It is more effective as a combined continuous and pulsed RFT than its other subtypes in treating primary TN patients. Moreover, RFT via a transverse puncture through the supraorbital foramen results in lesser inter- and post-procedural complications. Further, there is a slightly lesser incidence of post-procedural adverse effects and complications with RFT through the foramen rotundum. Besides, RFT, performed at a lower temperature of 65 degrees Celsius and a voltage between 64.51 and 79.29 volts, effectively provides pain relief and long-term patient satisfaction. RFT is safe and effective in patients over 60 with primary TN. Interestingly, it is also safe and effective in treating patients over 70 with poor fitness standards of Class II or higher. Despite these remarkable findings, there is still a substantial gap in the literature, specifically concerning the standardized protocol for temperature, voltage, and puncture methods of RFT. Despite the sufficient evidence of combined continuous and pulsed RFT’s superiority in efficacy and safety, most researchers still utilize either pulsed or continuous RFT. Studies vary in not only these aspects but also the patient cohorts. For instance, most researchers focus solely on evaluating RFT’s efficacy and safety in patients with primary TN, excluding a critical patient population suffering from secondary TN. Nevertheless, sufficient clinical evidence shows that RFT has come of age in treating primary TN patients. However, more extensive studies with large sample sizes of patients with primary and secondary TN with multiple trigeminal nerve affectation will significantly help standardize RFT protocol and its inclusion in the standard clinical practice in treating TN patients.

## Introduction and background

In the United States of America, about 15,000 people are diagnosed with trigeminal neuralgia (TN) every year [1]. Patients describe TN as sudden shooting pain in the facial areas innervated by trigeminal nerves. Irritation of any or all three branches of trigeminal nerves originating from the Gasserian ganglion, the ophthalmic division (V1), the maxillary division (V2), and the mandibular division (V3), can trigger TN. It can be unprovoked or may result from an innocuous stimulus, such as chewing [2]. However, such debilitating pain for a prolonged period can significantly affect patients’ quality of life (QOL) [3].

The first-line treatment of TN consists of sodium channel blockers such as carbamazepine or oxcarbazepine. Other medications used as an add-on in patients who do not respond to carbamazepine include lamotrigine, baclofen, pregabalin, and gabapentin [4]. But, if medications do not provide relief, the surgical approach is the next step in treating TN patients. Various surgical interventions, including microvascular decompression, partial sensory rhizotomy, glycerol rhizolysis, balloon compression, and gamma knife surgery, are in clinical practice [5]. However, with less favorable patient outcomes [6], recurrence rates [7,8], and lack of cost-effectiveness [9], there is a growing need for a more effective, minimally invasive, and cost-effective alternative.

Radiofrequency thermocoagulation (RFT) presents an effective, safe, and minimally invasive alternative to traditional procedures such as microvascular decompression [10]. Additionally, it is more cost-effective than other surgical approaches [11]. In the last decade and a half, various researchers have investigated the potential and established the effectiveness of RFT in treating patients with primary TN [12]. Yet, RFT is often under-utilized and is not the standard interventional procedure in treating TN patients who fail drug treatment(s). A few factors, such as no universal standardized RFT protocol and minimal or no awareness of its effectiveness and safety in specific cohorts of TN patients, for example, geriatric patients, may contribute to the lack of acknowledgment of RFT’s utility among neurosurgical practitioners. Therefore, this review aims to evaluate and highlight the advancement of RFT as a minimally invasive surgical tool in treating patients with primary TN and identify areas that need further improvement. In addition, it seeks to determine if RFT is safe and effective in elderly patients with TN. 

## Review

Literature search and study selection

**Figure 1:**
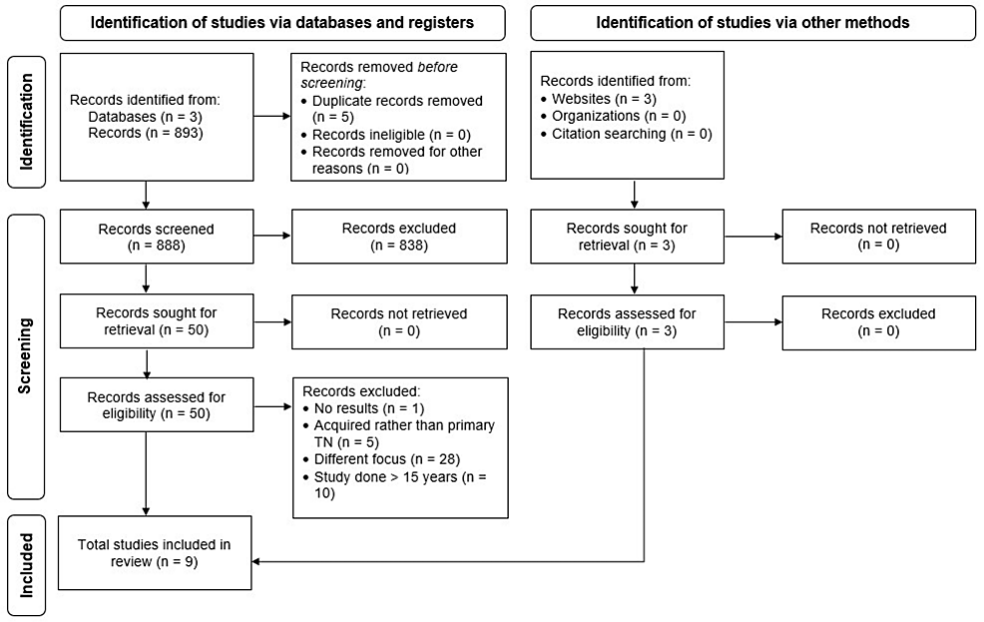
Literature search and study selection. This review utilized PubMed, Clinicaltrials.gov, and Embase databases for the literature search. By using the inclusion criteria, including articles written in English, and complete clinical trials with trigeminal neuralgia patients, the number of studies (n) was narrowed down to 9.

We conducted the literature search between July 2022 and March 2023 by following the Preferred Reporting Items for Systematic Reviews and Meta-Analyses (PRISMA) guidelines [13]. We utilized three databases: PubMed, ClinicalTrials.gov, and Embase. Furthermore, we expanded our search to three websites: Wiley Online Library, Pain Physician Journal, and Neural Regeneration Research. Figure 1 illustrates the PRISMA flowchart delineating the study selection and the total number of studies reviewed for this article. In addition to the inclusion and exclusion criteria, as mentioned in Table 1, we limited the search to the last 15 years and excluded preliminary clinical trials or trials that reported partial or no results. Also, we excluded studies not published in the English language. We included only relevant studies and assigned a level of clinical evidence as per the previous literature [14].

**Table 1:** Study selection criteria. Studies published in English from 2008 to 2023, focusing on patients with primary trigeminal neuralgia, and meeting the inclusion criteria, were included in the review.

Inclusion criteria	Exclusion criteria
Randomized clinical trials	Case series
Non-randomized clinical trials	Case reports
Prospective clinical studies	Meta-analysis
Double-blinded controlled studies	Systematic reviews
Single-blinded controlled studies	Narrative reviews
Retrospective studies	Preclinical studies
Observational studies	Commentaries

The key search terms were “Trigeminal Neuralgia,” “Trigeminal Neuralgia AND Radiofrequency Thermocoagulation,” “Trigeminal Neuralgia AND Combined Continuous And Pulsed Radiofrequency Thermocoagulation,” “Trigeminal Neuralgia AND Radiofrequency Thermocoagulation AND Puncture Method AND Puncture Site,” “Trigeminal Neuralgia AND Radiofrequency Thermocoagulation AND Temperature,” “Trigeminal Neuralgia AND Radiofrequency Thermocoagulation AND Voltage,” and “Trigeminal Neuralgia AND Radiofrequency Thermocoagulation AND Elderly Patients.”

Radiofrequency thermocoagulation and its subtypes

RFT is considered an effective, safe, and minimally invasive therapeutic method compared to other surgical interventions, such as microvascular compression, for treating patients with TN. The RFT procedure involves inserting an electrode that produces current into the tissues to cause ion mobility in the electric field, which has heat. This heat generation affects nerve(s) and, thus, prevents the pain signal transduction down the axons [10]. RFT only targets the afferent fibers of nociceptors, Aδ and C-type fibers, that modulate pain, as the Aα and Aβ-type fibers that conduct tactile neurofibrils are heat tolerant. Therefore, RFT does not impact them. Through targeting Aδ and C-type fibers, RFT provides pain relief in TN patients [15].

There are two types of RFT: pulsed radiofrequency thermocoagulation (PRFT) and continuous radiofrequency thermocoagulation (CRFT) [16]. PRFT provides an interval-based voltage to modulate pain conduction. PRFT involves the generation of 20 milliseconds of pulsed current with a silent period of 480 milliseconds at 42 degrees Celsius (°C) [10]. It is considered a safer technique but has a higher chance of recurrence. In contrast, CRFT is a percutaneous procedure involving continuous thermal ablation of the Gasserian ganglion, usually between 65°C and 80°C. Neurosurgeons can prefer CRFT as a surgical alternative due to its non-invasive nature compared to an invasive microvascular decompression. Moreover, CRFT has a lower risk of cranial infection, cerebrospinal fluid leak, and hearing loss [16]. Traditionally, RFT involves a C-arm guided approach. C-arm machines enable surgeons to visualize the puncture site and location of the affected trigeminal nerve(s) through X-ray technology [17]. However, research has shown that computed tomography (CT)-guided RFT is more accurate than a C-arm-guided RFT [18].

RFT progressing to combined continuous and pulsed radiofrequency thermocoagulation

The rapid evolution of RFT from its basic form, PRFT or CRFT, to an innovative and relatively advanced form in combined continuous and pulsed radiofrequency thermocoagulation (CCPRF) is noteworthy. In the last decade, researchers, for example, Elawamy et al. (2017), have demonstrated the effectiveness of CCPRF in treating TN patients [19]. They randomly assigned 43 patients into three groups in a prospective study. In the first group, 11 patients received C-arm guided PFRT at 45 volts (V), with a pulse width of 10 milliseconds (ms) and a pulse frequency of 4 Hertz (Hz) for 10 minutes at 42°C. In the second group, 12 patients were given C-arm guided CRFT continuously, without any intervals, for 4.5 minutes at 75°C. Finally, in the third group, 20 patients received CCPRF, a combination of C-arm guided PRFT and C-arm guided CRFT. They were first treated with PRFT at 45 V, with a pulse width of 10 ms and a pulse frequency of 4 Hz for 10 minutes at 42°C and were subsequently administered CRFT continuously, without any intervals, for 4.5 minutes at 60°C. The study protocol required assessing patients for pain using a visual analog scale (VAS), satisfaction, and dose of concurrent medication for pain management at baseline and post-RFT intervention after seven days, one month, six months, and 24 months. Furthermore, the researchers documented complications, facial weakness, numbness, and other adverse effects [19].

The study findings showed that patients who underwent CCPRF had the most significant pain relief. In the CCPRF group, 80% of patients reported pain relief (p = 0.004) after six months, 85% reported patients pain relief after 12 months (p = 0.001), and 90% experienced pain relief at 24 months (p = 0.001) compared to 75% after six and 12 months and 50% after 24 months in the PRFT group and 18.2% at after six months, 9.1% after 12 months, and 0% after 24 months in the CRFT groups, respectively. Regarding quantifying pain using a VAS, there were no significant differences in the patient baseline scores across the three groups. However, patients in the CCPRF group rated the pain at 0 after six, 12, and 24 months, respectively. On the other hand, in the PRFT group, patients rated the pain at 0 after six months, 0.833 ± 0.28 after 12 months, and 1.83 ± 0.36 after 24 months. Similar to the PRFT group, interestingly, patients in the CRFT group reported a gradual increase in pain with a score of 0 after six months, 1.18 ± 0.17 after 12 months, and 2.63 ± 0.14 after 24 months, respectively. The gradual increase in patients’ VAS scores obtained after 6, 12, and 24 months in PRFT and CRFT groups indicates a TN recurrence compared to no recurrence in patients in the CCPRF group [19].

There were no significant differences (p = 0.695) in the carbamazepine dose among the three groups at baseline. However, it is worth mentioning that patients in the PRFT group still needed carbamazepine 24 months after the procedure, albeit 22.22 ± 6.6 milligrams per day, compared to patients in CRFT and CCPRF groups, where patients did not need the medication. Another crucial aspect of the study was patient satisfaction post-intervention. Patients undergoing CCPRF expressed higher satisfaction than those who underwent PRFT and CRFT procedures after one (p = 0.033) and six months (p = 0.033), respectively. Further, patients in the CCPRF group experienced the least complications (20%) compared to patients in CRFT and PRFT groups, with 45.5% and 25% of patients experiencing complications post-intervention, respectively. About 18% of patients reported facial numbness and weakness after undergoing CRFT. However, only 9% reported paresthesia after undergoing CCPRF. The researchers postulated that thermal damage to myelinated and unmyelinated nerves in the brain resulted in complications experienced by the patients across groups [19].

Although the study’s sample size was limited, the researchers showcased the effectiveness and safety of CCPRF in treating TN patients. Their data also indicated a low TN recurrence probability with CCPRF. However, more clinical trials with larger sample sizes are required to explore its potential as a surgical option with a lower-to-zero recurrence rate [19].

Further building on the remarkable findings of Elawamy et al. (2017) and refining the technique, Ding et al. (2018) investigated if CCPRF is comparable to or more effective than CRFT in treating primary TN patients [20]. Instead of performing traditional C-arm-guided-RFT, they utilized CT guidance to perform RFT. In a randomized clinical trial, the researchers randomly assigned 80 patients to two groups: 40 patients in the CRFT group and 40 in the CCPRF group. The study patients, older than 30 years, were diagnosed with primary V2, V3, and V2 plus V3 TN who had unsuccessful treatment with carbamazepine. Researchers performed CT-guided RFT through the foramen ovale. Patients in the CRFT group received CRFT at 68°C for three minutes under CT guidance. On the contrary, in the CCRPF group, patients first received PRFT at 42°C for 10 minutes and then immediately underwent CRFT at 68°C for three minutes under CT guidance. The study protocol assessed pain based on VAS scores, which helped determine the degree of pain relief indexed as a surgical efficiency rate. In addition, the study recorded any post-procedural complications, including bleeding, nausea, vomiting, headache, facial numbness, decreased corneal reflex, and masticatory muscle weakness. The patient follow-up time points were one day, a month, six months, a year, and two years post-surgical intervention [20].

The study results demonstrated a significant decrease in patients’ VAS scores in both groups compared to baseline (pre-surgery) (p < 0.05). However, between the two groups, patients in CCPRF reported significantly lower VAS scores at each follow-up up to two years post-procedure than patients in the CRFT group (p < 0.05). Additionally, patients in the CCPRF group had a remarkably greater efficiency rate of 97.5% at six-month, one-year, and two-year follow-up post-procedure than patients in the CRFT group who had an efficiency rate of 85% at the same follow-up timepoints post-procedure (p < 0.05) [20].

Neither group differed in the types and number of post-procedural complications or adverse effects. However, patients in the CCPRF group had a remarkably shorter recovery time from such complications than those in the CRFT group (p < 0.05). For example, in the CCPRF group, patients who experienced facial numbness recovered within 2.23 ± 1.02 months compared to patients in the CRFT group who took 3.12 ± 1.21 months to recover (p > 0.05). Similarly, patients who developed masticatory muscle weakness in the CCPRF group recovered within 3.42 ± 0.98 months compared to 4.33 ± 1.54 months in the CRFT group (p < 0.05). Notably, the recurrence rate was profoundly lower in the CCPRF group (5%) compared to the CRFT group (20%) (p < 0.05) [20].

With this randomized clinical trial with a moderately larger sample size, Ding et al. (2018) demonstrated that CCPRF is more effective in treating patients with V2, V3, and V2 plus V2 primary TN than CRFT. Moreover, they refined the technique by utilizing CT-guided CCPRF and CRFT and showcased that CT-guided CCPRF and CRFT were robust and could produce effective patient outcomes. However, more research directly comparing the C-arm-guided CCPRF and CT-guided CCPRF would establish and further elucidate the precision, effectiveness, and robustness of CT-guided CCPRF and its impact on long-term outcomes in patients with TN [20].

Emerging approaches to puncture method and puncture sites in the RFT

Evidence illustrates that the puncture method in RFT performed can impact the procedure's effectiveness and, thus, patient outcomes. Wang et al. (2021), in a randomized, longitudinal prospective study, analyzed two different puncture methods to carry out RFT [21]. They investigated vertical puncture and transverse puncture through the supraorbital foramen. Their study recruited 57 patients diagnosed with V1 TN and randomized them into vertical and transverse puncture groups. They determined whether a difference in RFT puncture methods could impact post-surgical short-term complications such as facial pain, numbness, long-term adverse effects, and pain-free survival rates. The study protocol involved inserting a 22-gauge radiofrequency puncture needle into the supraorbital foramen under CT guidance. In the vertical puncture group, surgeons inserted the needle vertically into the supraorbital foramen. In the transverse groups, they inserted the needle laterally from the skin near the supraorbital foramen. The puncture was considered complete once the needle tip reached the supraorbital foramen. Upon puncture completion, the surgeons fixed the needle, pulled out the needle core, injected propofol as an anesthetic agent, and then inserted the radiofrequency electrode and rendered CRFT at 95°C for two minutes [21].

The researchers reported that patients experienced remarkably lesser post-procedure facial pain, indexed as VAS scores, in the transverse puncture group than patients in the vertical puncture group. Patients in the transverse puncture group had significantly lower VAS scores, 1.07 ± 0.66 at six months (p < 0.001), 0.86 ± 0.85 at one year (p < 0.001), and 1.11 ± 1.34 at two years (p < 0.001), post-procedure than patients in the vertical puncture group, 2.34 ± 1.47 at six months, 2.55 ± 1.52 at one year, and 2.79 ± 1.82 at two years. Patients in both groups did not experience facial numbness. The other short-term post-procedure complications included ulcers and diplopia; both were in the vertical puncture group. One patient developed a puncture-point ulcer, and another suffered optic nerve damage. The needle slipped accidentally into the back of the eyeball during RFT performed through the vertical puncture method in the supraorbital foramen, resulting in optic nerve damage and hence, diplopia [21].

The study findings also showed that patients in the transverse puncture group had significantly better long-term outcomes than those in the vertical puncture group. The long-term effects measured as pain-free survival rates were 93% at one day (p < 0.05), 82% at six months (p < 0.05), 75% at one year (p < 0.05), and 64% at two years (p < 0.05) post-procedure in the transverse puncture group compared to 72% at one day, 45% at six months, 41% at one year, and 38% at two years post-procedure in the vertical puncture group. However, the study’s primary limitation was that the researchers excluded cohorts with V2 and V3 TN. Also, the sample size could have also been larger, allowing for generalizing the study findings in the larger clinical context. Notwithstanding, the study demonstrated that RFT performed through the transverse puncture in the supraorbital foramen could be significantly effective in treating patients with V1 TN [21].

Further evaluating RFT effectiveness in patients with V2 plus V3 TN, other researchers studied RFT foramen rotundum and foramen ovale. For example, Zeng et al. (2020) studied CT-guided RFT targeting V2 and V3 TN through foramen rotundum and ovale, respectively, in patients with V2 plus V3 TN and sought to minimize corneal ulcers, optic damage, and intracranial hemorrhage, as seen with traditional RFT through the supraorbital foramen targeting Gasserian ganglion. In a more extensive, prospective, randomized controlled trial, they enrolled 102 patients with unilateral V2 plus V3 TN who failed to respond to any drug treatment and did not have a previous surgical intervention, such as microvascular compression [22].

The study protocol randomized patients into the peripheral radiofrequency (PRF) group and the ganglionic radiofrequency (GRF) group, with 51 patients in each group. The puncture sites for RFT in the PRF group were foramen rotundum and foramen ovale, and patients received CRFT to V2 and V3, respectively, separately at 50°C for three minutes. The temperature varied between 50°C and 75°C based on patients’ tolerance. In the GRF group, the puncture site was the foramen ovale, and the rest of the procedure was similar to that performed in the PRF group. The Barrow Neurological Institute (BNI) pain scale assessed the patients’ pain immediately after surgery and postoperatively at two years. The BNI scores were categorized into different classes: Class I - II, if a patient required no pain medication(s) and experienced no pain or only occasional pain; Class III - V, if a patient needed pain medication(s) or medication(s) failed to control pain, and a patient experienced elevated levels of pain or severe pain. The study recorded the patient outcomes by assessing the immediate effective rate, the postoperative effective rate (at two years), the postoperative recurrence rate (at two years), and the number of complications. The immediate effective rate referred to pain relief with patients’ BNI scores in Class I or II 24 hours after surgery. The effective recurrence rate was patients’ relapse to previous worsening pain levels after accomplishing higher pain relief postoperatively [22].

The study findings showed that patients in the PRF group had a significantly greater immediate effective rate of the V2 branch, evidenced by the PRF group having an effective rate of 50% compared to the GRF group at 41% (p = 0.038). Interestingly, there was no significant difference in the effective rates between the two groups postoperatively after two years, except for the effective rate of the V3 branch, which was greater in patients in the GRF group than PRF group (p = 0.0002), consistent with the V3 branch’s significantly lower recurrence rates (4%) in the GRF group compared to PRF group (8%) (p = 0.016) postoperatively after two years. However, patients in the GRF group suffered from more complications, albeit short-term, than the PRF group. For example, two patients developed numbness in the V1 innervation area, and one had a corneal ulcer. But patients in the PFR group experienced more facial swelling, which the researchers attributed to two puncture sites compared to just one in the GFR group. Patients in neither group experienced intracranial hemorrhage or infection, deafness, or orbital apex syndrome. The study’s crucial limitation that might make surgeons apprehensive about this approach was the temperature, ranging between 50°C and 75°C based on a patient’s tolerance level instead of utilizing a fixed or standardized temperature to perform RFT. Investigating if a standardized or lower temperature range could yield similar results in this patient cohort would be interesting and warrant more research. Nevertheless, the study findings indicated that RFT through foramen rotundum and ovale is safer and more effective in treating patients with V2 plus V3 TN. However, RFT targeting Gasserian ganglion through foramen ovale could lead to post-surgical complications, including corneal ulceration, but might be better at preventing V3 TN relapse [22].

To further establish whether CT-guided RFT through the foramen rotundum is more effective and safer than foramen ovale in treating patients with V2 TN specifically, Ding et al. (2021) in a prospective and randomized controlled trial enrolled 70 patients with V2 TN who failed the standardized drug treatment and did not have a secondary TN, such as due to intracranial space-occupying lesions. They randomized patients into the foramen rotundum group (FRG) and the foramen ovale group (FOG), with 35 patients in each group. The puncture site in the FRG group was the foramen rotundum, and patients underwent CRFT to V2 for three minutes at a temperature ranging between 50°C and 68°C. In the FOG group, the puncture site was the foramen ovale, and the CRFT treatment to V2 was similar. The VAS scale assessed patients’ pain, and the Medical Outcomes Study 36-Item Short-Form Health Survey evaluated patients’ QOL, assessing both physical and mental states. The total efficacy rate was categorized into excellent, effective, and ineffective. In addition, it was rated excellent if pain, numbness, and hyperalgesia did not exist, effective if pain and numbness were significantly reduced, and ineffective if symptoms were not improved. The study further documented short-term post-procedure complications, including facial hematoma, headache, nausea, vomiting, and long-term adverse effects, such as facial numbness, decreased corneal reflex, masticatory muscle weakness, and recurrence rates. The assessment time points included the preoperative (baseline) and postoperative at one week, two weeks, one month, three months, six months, and one year [23].

The study findings showed that patients in both groups experienced considerable pain relief, evidenced by significantly reduced VAS scores postoperatively compared to baseline (p < 0.05). However, the two groups had no significant difference in the postoperative VAS scores at various assessment time points. Moreover, there was a remarkable improvement in patients’ QOL in both groups postoperatively compared to baseline (p < 0.05). But for patients in the FRG group, the physical and mental state scores were significantly higher after one week, two weeks, and one month than those in the FOG group (p < 0.05). However, patients in the FOG group reported gradually increased scores after three months. Subsequently, the two groups had no significant difference in QOL scores at six months and one-year follow-ups. Notably, the two groups did not differ statistically in the total efficacy rates, with patients in the FRG group reporting an 80% total efficacy rate compared to 74.3% in the FOG group. The most remarkable findings were the total incidence of complications. The total incidence of complication was 20% in the FRG group compared to 62.9% in the FOG group (p < 0.05). Despite the study’s limited sample size, its findings demonstrated that RFT through the foramen rotundum and ovale is equally effective in treating patients with V2 TN. Yet, RFT through the foramen rotundum might be slightly advantageous due to its lesser postoperative total incidence of complications [23].

Optimizing the RFT temperature

An integral part of RFT is the temperature. While most researchers have carried out RFT at a temperature ranging from 60°C to 95°C, unfortunately, there has not been a consensus on RFT temperature. One group of researchers, Yao et al. (2016) set out to study if a specific temperature used during RFT can impact patient outcomes and, if so, what a minimum effective temperature could be [24]. In a prospective study following up on patients up to five years after RFT intervention, they enrolled 62 patients diagnosed with V2, V3, and V2 plus V3 TN and assigned them to two groups. Patients in the first group received CRFT at 68°C for three minutes through the foramen ovale. Patients in the second group received CRFT at 75°C for three minutes through the foramen ovale. The study evaluated pain relief, pain-free rate, complications, and patient satisfaction postoperatively after one year, three, and five years [24].

The study findings showed that patients who received RFT at 75°C had significantly higher pain relief (p < 0.05) than those who received RFT at 68°C. However, the two groups had no statistical difference in the pain-free rates after one year, three years, and five years. However, patients who underwent RFT at 75°C had significantly more complications following the surgical intervention than those who underwent RFT at 68°C. For example, 79% of patients experienced facial numbness after undergoing RFT at 75°C compared to 12.9% of patients who had RFT at 68°C group (p < 0.05). Additionally, masticatory atonia was seen in 16 patients in the 75°C group, whereas only three patients experienced it in the 68°C group (p < 0.05). Most notable were the patient satisfaction findings. Patients in the 68°C group had significantly higher patient satisfaction, with 90.3% at one year, 66.2% at three years, and 63.6% at five years, compared to those in the 75°C group, with 69% at one year, 56.8% at three years, and 50.6% at five years (p < 0.05) [24].

Despite having a robust study protocol measuring the impact of temperature with clearly defined patient outcomes such as pain relief, pain-free rate, and patient satisfaction in the V2, V3, and V2 plus V3 TN patient cohort, the study was limited in its scope. The researchers only employed RFT at two specific temperatures: 68°C and 75°C. The study should have had more treatment groups with RFT performed in a wide range of specific temperatures. It would have allowed us to ascertain better the minimum effective and maximum temperatures at which RFT could be performed effectively and safely. Optimizing the therapy for its applicability to patients with different types of TN could prove vital [24].

Improving the RFT voltage

Having previously demonstrated that patients could tolerate an adjustment in voltage during PRFT from 27 V to 50 V, Fang et al. (2014) sought to determine if a voltage over 60 V was effective and safe in a randomized control trial in treating patients with primary TN. The researchers randomly assigned 53 patients into two groups: the standard-voltage group and the high-voltage group. In the standard-voltage group, 27 patients received PRFT through foramen ovale at 42°C for four minutes at 2 Hz intervals with voltage ranging from 30.93 V to 41.81 V. On the other hand, in the high-voltage group, 26 patients received PRFT in similar conditions but at a higher voltage ranging from 64.51 V to 79.29 V. The study protocol involved adjusting voltages based on patient tolerance. The patients in the standard-voltage group received an average of 36.37 V ± 5.44 V, whereas patients in the high-voltage group received an average of 71.90 V ± 7.39 V without discomfort [25].

The study outcomes included assessing patients’ pain levels using the Numeric Rating Scale to determine the effective rate, carbamazepine dose, and surgical complications at one day, one week, two weeks, one month, two months, three months, six months, and one year postoperatively. The study findings showed that patients in the high-voltage group had a significantly higher effective rate of 69% than patients in the standard-voltage group, who had an effective rate of 41% at one, three, and six months postoperatively (p = 0.037). Notably, patients in the high-voltage group continued to have an effective rate of 69% at one-year postoperatively. In contrast, patients in the standard-voltage group reported a 19% effective rate at one-year postoperative (a remarkable 22% decrease from six months postoperatively). The two groups had no statistical difference regarding carbamazepine dose, its discontinuation, and post-surgical complications. There were no serious adverse effects noted in either group [25].

Although the study sample size was relatively smaller, the researchers successfully showed that high-voltage RFT between 64.51 V and 79.29 V could effectively treat patients with primary TN. Furthermore, it displayed that a lower temperature of 42°C could also be effective with no complications at various high voltages. However, more studies with larger sample sizes, including patients with different types of TN, would further establish these findings and help broadly apply them to clinical practice [25].

RFT's effectiveness and safety in older TN patients

There is evidence that TN incidence increases with age [26]. Yet there is insufficient literature on whether RFT is safe for geriatric patients. Research endeavors led by Lai et al. (2011) showed that CRFT is effective and safe in older patients [27]. In a large clinical trial, Lai et al. (2011) recruited 852 patients and assigned them to two groups based on their ages, with 502 patients aged 60 years or older in the first group and 350 patients below 60 years in the second group. Study patients had V1, V2, V3, V1 plus V2, V2 plus V3, and V1 plus V2 plus V3 primary TN. The study protocol utilized VAS scores to assess pain before the procedure and at discharge, and Number Rating Scale (NRS) scores at one-, two-, and three-year follow-up post-procedure. It also recorded post-procedure complications or adverse effects and determined the QOL by utilizing QOL-EuroQol-5D questionnaire Patients in both groups underwent CT-guided CRFT through foramen ovale at 65°C - 75°C for three minutes [27].

The study findings showed that patients in both groups had significant pain relief (calculated from VAS scores) post-procedure at discharge. However, there was no statistical difference between the two groups, with 94.9% pain relief in patients who were ≥ 60 years and 93.7% pain relief in patients who were < 60 years. Similarly, there was no remarkable difference between the two groups at a three-year follow-up, with 96.8% of patients reporting pain relief (calculated from NRS scores) who were ≥ 60 and 98.6% reporting pain relief who were < 60. Moreover, patients in both groups significantly improved their QOL at three-year follow-up compared to pre-procedure (baseline) (p < 0.05). However, there was no significant difference in patients’ QOL scores between the two groups at the same follow-up timepoint, with 89.26 as the average QOL score in patients aged 60 years and older and 90.53 as the average QOL score in patients who were below 60 years [27].

Most notably, neither group reported any severe complications or adverse effects post-procedure. Of patients who were 60 or above, two had decreased visual acuity (0.4%), another two had masseter dysfunction (0.4%), and one patient had a decreased gustatory sensation (0.2%). All patients in this age group and those below 60 years experienced trigeminal hypesthesia. Additionally, of patients who were below 60 years, one patient developed corneal ulcers (0.3%), one experienced palpebral frontalis ptosis (0.3%), and another had a decreased gustatory sensation (0.3%). However, there was no record of these patients’ recovery rate from these minor complications. The study results illustrated CRFT as a safe and effective procedure in older and younger patients with primary TN. The study’s large sample size (consisting of TN patients with multiple trigeminal branches affected) and its robust protocol provided validity to these findings. In addition, it laid the groundwork for other researchers, such as Tang et al. (2014), to further investigate its safety and effectiveness in older TN patients [27].

In a comparative analysis, a research group helmed by Tang et al. (2014) evaluated whether CRFT is effective in primary TN patients aged 70 and older. In addition, they also investigated if CRFT could benefit such patient cohorts with poor fitness status categorized as Status II and higher per the American Society of Anesthesiologists Classification System. The study recruited 304 patients assigned to two distinct groups based on the temperature of CRFT. The first group had 37 patients who underwent CT-guided CRFT through foramen ovale, performed at 80°C or higher for two to three minutes. However, the second group had 267 patients who underwent a similar CRFT procedure but performed at 75°C or lower. One hundred eighty-seven patients had Class II fitness status, and only 67 had Class III fitness status [28].

Post-CRFT, patients in both groups had significant pain relief (100%) at discharge. On follow-up after one, three, five, and ten years, the pain relief was 85%, 75%, 71%, and 49%, respectively. There was no statistical difference in pain relief between the two groups, including those with Class II or higher fitness status. Patients in both groups experienced post-procedural complications, albeit minor, such as facial numbness, masseter weakness, corneal inflammation, hearing loss, droopy eyelid, mouth’s decreased range of motion, and dysesthesia. There was no statistical difference between the two groups concerning the types and incidence rate of post-procedural complications, except for dysesthesia. Patients in the higher temperature group (80°C or higher) had a more profound incidence rate of dysesthesia than those in the lower temperature group (75°C or lower). Most notable was the lack of additional morbidity and mortality in patients with physical status II or higher following CRFT intervention [28].

Tang et al. (2014) demonstrated that CRFT was not only effective in primary TN patients who were 70 and older but was significantly beneficial in those who had poor fitness standards of Class II or higher. The distinguishing feature of their study was the patient follow-up from pre-CRFT (baseline) up to 10 years post-CRFT. The study’s longer duration made it possible to comprehensively evaluate long-term patient outcomes of CRFT regarding its effectiveness and safety in a vulnerable patient cohort with TN aged 70 or higher [28].

Although the last decade and a half have seen RFT making significant improvements in evaluating the efficacy of combining different RFT types, innovation in puncture method and site, and exploration and optimization of its technical aspects such as temperature and voltage (see Table 2), there is significant room for more research endeavors to standardize the RFT in treating patients with TN. One of the critical areas of research is more extensive sample sizes with patient cohorts who have not only V1, V2, and V3 trigeminal nerve affectation but also have different degrees and mixtures of trigeminal nerve blockade. In addition, variation in patient outcomes parameters, lack of comprehensive assessments, and absence of long-term follow-ups across study protocols pose a significant challenge for standardizing the RFT therapy in treating patients with TN. Another often underrepresented but crucial area requiring more research is RFT effectiveness in treating refractory patients who have either failed pharmacotherapy or have undergone unsuccessful surgical intervention, such as microvascular decompression. Finally, there is a considerable gap in the literature assessing RFT's utility in treating patients with secondary TN. Nevertheless, as highlighted in the review, adequate clinical evidence indicates RFT can be massively beneficial in treating primary TN patients and offers them a less-invasive, safer option.

**Table 2:** Summary of the reviewed studies highlighting the advancement of radiofrequency treatment (RFT) in the last decade and a half. The studies mentioned in this table met this systematic review’s inclusion criteria. Key study outcomes depicted the progression of RFT to combined continuous and pulsed radiofrequency thermoregulation (CCPRF), RFT’s advancements regarding the puncture method and puncture sites, RFT’s optimization for temperature and voltage, and its effectiveness and safety in older primary trigeminal neuralgia patients. *°*C, degrees Celsius; CRFT, continuous radiofrequency thermocoagulation; FOG, foramen ovale group; FRG, foramen rotundum group; GRF, ganglionic radiofrequency; *Hz*, Hertz; ms, milliseconds; PRF, peripheral radiofrequency; PRFT, pulsed radiofrequency thermocoagulation; QOL, quality of life; VAS, visual analog scale; V, volts.

Authors	Study Design	Level of Evidence	Sample Size	RFT Subtype	Procedure Specifications (Temperature, Voltage, and Time)	Key Findings
Progression of RFT to CCPRF						
Elawamy et al. (2017) [19]	Randomized controlled trial	I	43	PRFT, CRFT, and CCPRF	PRFT: 45 V with a pulse width of 10 ms and 4 Hz for 10 minutes at 42°C; CRFT: 4.5 minutes at 75°C; CCPRF: Combined 45 V with a pulse width of 10 ms and 4 Hz for 10 minutes at 42°C and 4.5 minutes at 75°C.	CCPRF group experienced the most significant pain relief following surgery (p = 0.0001). There were no significant differences in VAS scores, but the CCPRF group reported the greatest improvement. PRFT group still needed carbamazepine for months following the procedure. CCPRF group reported the highest satisfaction (p = 0.0333) and the least number of complications.
Ding et al. (2018) [20]	Randomized controlled trial	I	80	CRFT and CCPRF	CRFT: 68°C for three minutes; CCPRF: 42°C for 10 minutes and 68°C for three minutes	CCPRF group reported lower VAS scores (p < 0.05). CCPRF group had a greater efficiency rate (p < 0.05). CCPRF had a shorter recovery time and lower recurrence rate following surgery (p < 0.05).
Advancement in Puncture Method and Puncture Sites in RFT						
Wang et al. (2021) [21]	Randomized controlled trial	I	57	CRFT	95°C for two minutes	VAS scores were lower in the transverse puncture group than in the vertical puncture group (p < 0.001). Significant long-term pain relief in the transverse puncture group (p < 0.05).
Zeng et al. (2020) [22]	Randomized controlled trial	I	102	CRFT	50°C for three minutes for the PRF group and 50°C to 75°C based on tolerance for the GRF group	PRF group had a greater immediate effective rate of the V2 branch (p = 0.038) and the V3 branch after two years (p = 0.0002). Lower recurrence rate in the GRF group in the V3 branch (p = 0.016). Patients in the GRF group suffered more postoperative complications.
Ding et al. (2021) [23]	Randomized controlled trial	I	70	CRFT	50°C to 68°C for three minutes	Both the FOG and the FRG groups had profoundly reduced VAS scores. They had a remarkable improvement in their QOL (p < 0.05). The FRG group had fewer postoperative complications (p < 0.05).
Optimization of the RFT Temperature						
Yao et al. (2016) [24]	Randomized controlled trial	I	62	CRFT	68°C for three minutes and 75°C for three minutes	The patients in the high-temperature group (75°C) had higher pain relief compared to those in the low-temperature group (68°C) (p < 0.05). The high-temperature group (75°C) had more postoperative complications (p < 0.05). However, patients in the low-temperature group (68°C) had higher satisfaction rates (p < 0.05).
Improvements in the RFT Voltage						
Fang et al. (2014) [25]	Randomized controlled trial	I	60	PRFT	42°C for four minutes at 2 Hz with 30.93 V to 41.81 V and 42°C for four minutes at 2 Hz with 64.51 V to 79.29 V	The high-voltage group had a higher effective rate than the standard-voltage group (p = 0.037). There were no serious adverse effects in either group.
RFT Effectiveness and Safety in Older TN Patients						
Lai et al. (2011) [27]	Non-randomized controlled trial	II	852	CRFT	65°C to 75°C for three minutes	The patients in the older group (patients aged ≥ 60) and the younger group (patients below 60) had significant pain relief. However, the two groups had no statistical difference in the degree of pain relief. Both groups had improved QOL at the three-year follow-up (p < 0.05). Neither group reported severe complications.
Tang et al. (2014) [28]	Non-randomized controlled trial	II	304	CRFT	80°C or higher for two to three minutes and 75°C or lower	Patients aged 70 and older had 100% pain relief at discharge in both the 80°C group and the 75°C group. There was no statistical difference between the two groups concerning the post-procedural complications, except for dysesthesia observed in the 80°C group. CRFT was beneficial in patients with poor fitness standards of Class II or higher.

## Conclusions

In the last decade and a half, RFT has presented itself as a safe, effective, minimally invasive surgical option for treating primary TN patients. This systematic review highlights the advancement of RFT from its primitive C-arm guided PRFT and CRFT subtypes to its more robust, highly effective, and safer CT-guided CCPRF form. Moreover, it showcases that RFT is highly effective via the transverse puncture and through the foramen rotundum at 65°C and between 64.51 V and 79.29 V. Additionally, it showcases that RFT is safe and effective in patients over 60 and those with poor fitness standards with Class II or higher. However, the lack of uniformity in the RFT protocols across studies, smaller sample sizes, and exclusion of TN patients with multiple branch affectation and secondary TN augur the need for more extensive research to standardize RFT and its subsequent incorporation in the standard clinical practice. Notwithstanding, substantial clinical evidence indicates that RFT is safe and effective in patients with primary TN and has come a long way as a treatment procedure for such patient cohorts.
